# Endogenous Biomarkers Analysis and False-Negative Results for SARSCov2 Using two Commercial RT-PCR Diagnostic Kits

**DOI:** 10.30699/ijp.2025.2053609.3417

**Published:** 2025-11-11

**Authors:** Mahdieh Khoshakhlagh, Toktam Dehghani, Alaleh Alizadeh, Mojtaba Meshkat, Samaneh Abolbashari, Aida Gholoobi, Fatemeh Ghaemi, Zahra Meshkat

**Affiliations:** 1 Metabolic Syndrome Research Center, Mashhad University of Medical Sciences, Mashhad, Iran; 2 Department of Biochemistry, Faculty of Medicine, Mashhad University of Medical Sciences, Mashhad, Iran; 3 Department of Microbiology and Virology, Faculty of Medicine, Mashhad University of Medical Sciences, Mashhad, Iran; 4 Student Research Committee, Faculty of Medicine, Mashhad Branch, Islamic Azad University, Mashhad, Iran; 5 Department of Community Medicine, Faculty of Medicine, Mashhad Medical Sciences, Islamic Azad University, Mashhad, Iran.; 6 Antimicrobial Resistance Research Center, Mashhad University of Medical Sciences, Mashhad, Iran; 7 Medical Genetics Research Center, Mashhad University of Medical Sciences, Mashhad, Iran

**Keywords:** COVID-19, Real-time PCR, internal control, false-negative diagnoses, respiratory tract samples

## Abstract

**Background & Objective:**

Real-time PCR is widely used to detect SARS-CoV-2, the virus responsible for COVID-19, but false-negative results can occur even with internal controls. This study aimed to investigate the impact of using alternative internal control materials on the accuracy of SARS-CoV-2 detection kits.

**Methods:**

Between December 2021 and January 2022, 162 respiratory tract samples were collected from patients with suspected COVID-19 at Ghaem Hospital in Mashhad, IR Iran. Samples were initially tested with the Pishtaz Teb kit, which uses DNA internal control, and then negative samples were retested with the Geneova kit, which uses a RNA internal control. Positive and negative controls were consistently used to validate the results.

**Results:**

After retesting with the Geneova kit, only one patient out of 162 negative samples was positive for SARS-CoV-2. The Pishtaz Teb and Geneova controls consistently produced the expected results, but the Geneova internal control matched the Pishtaz Teb control in only 44% of cases. The higher threshold cycle value for Geneova internal control suggested RNA degradation during the experimental period.

**Conclusion:**

Proper quality control measures are crucial for accurate SARS-CoV-2 detection. The study highlights the importance of selecting reliable diagnostic kits with high sensitivity and specificity to reduce false-negative results, particularly in cases with a low viral load or early stages of the disease. The internal RNA control can detect RNA degradation and help identify false-negative diagnoses, leading to better disease control and management. Further research is needed to improve the accuracy of COVID-19 diagnostic tests.

## Introduction

The severe acute respiratory syndrome coronavirus 2 (SARS-CoV-2) was first identified in China in 2019 and is responsible for the coronavirus disease 2019 (COVID-19) pandemic (1-3). Common symptoms of COVID-19 include fever, cough, headache, myalgia, and fatigue, and the virus is highly transmissible through contact and respiratory droplets (4, 5). Symptoms of COVID-19 typically appear 1-14 days after exposure to the virus and may even last up to 24 days (6-8). According to data from the World Health Organization, as of 28 February 2023, there have been 758,390,564 confirmed cases of COVID-19 worldwide, with 6,859,093 deaths (9).

The SARS-CoV-2 virus has a genetic material that is composed of linear, positive-sense, single-stranded ribonucleic acid (RNA) and is 29,903 base pairs in length. The viral RNA is encapsulated by the nucleocapsid protein (N), which is one of the four structural proteins that include the spike (S), envelope (E), and membrane proteins (M) (10). The viral genome also encodes several nonstructural proteins (nsps), including the RNA-dependent RNA polymerase (RdRP) (nsp12), papain-like protease (PLpro) (nsp3), and coronavirus main protease 3-chymotrypsin-like protease (3CLpro) (nsp5), all of which play crucial roles in viral replication (11, 12).

The two diagnostic test types that are important for patient management and disease control are molecular or nucleic acid amplification tests (like polymerase chain reaction (PCR) tests) that identify viral RNA, and antigen tests that detect viral proteins (such as nucleocapsid or spike proteins). Serology tests can identify host antibodies in response to infection, vaccination, or both, but they only offer limited information about infection 1-2 weeks after the symptoms begin and are best used for monitoring purposes (13). The tests available to detect SARS-CoV-2 antibodies from blood samples include lateral-flow immunoassays (LFIAs), enzyme-linked immunosorbent assays (ELISA), or chemiluminescent immunoassays (CLIA). Real-time reverse-transcription PCR (RT-PCR) is used as the gold standard method for analyzing respiratory tract samples, while molecular or nucleic acid amplification tests can diagnose acute infections (14, 15).

In addition to imaging modalities (16), inflammation parameters are an important diagnostic tool in identifying COVID-19 patients. Blood sugar levels, urea, creatinine, alanine aminotransferase, aspartate aminotransferase, alkaline phosphatase, bilirubin total, bilirubin direct, c-reactive protein (CRP), lactate dehydrogenase(LDH), and hematological parameters such as red blood cells(RBC), white blood cells (WBC), Platelet(PLT), prothrombin time (PT), partial thromboplastin time (PTT), international normalized ratio (INR), and erythrocyte sedimentation rate (ESR) are commonly measured in COVID-19 patients (17). These parameters identify inflammation and other associated abnormalities in COVID-19 patients (18). CRP and ESR levels are particularly useful in identifying pneumonia and severe disease patients as they are correlated with lung lesions (19). ESR levels have been shown to increase in patients with severe COVID-19 disease and also, they are predictive of mortality (20). Other parameters such as blood sugar levels, urea, creatinine, and liver function tests can help identify organ damage in COVID-19 patients. Hematological parameters such as RBC, WBC, and PLT can also be used to identify abnormalities associated with COVID-19 (21). Overall, inflammation parameters play an important role in the diagnosis and management of COVID-19 patients (22).

Despite the high sensitivity of PCR-based tests for COVID-19, false-negative results can occur (23). Studies have reported false-negative results for SARS-CoV-2 from respiratory samples ranging from 1 to 30%. Several factors can contribute to false-negative results, including inadequate specimen collection, testing conducted too early in the course of the disease, limited analytic sensitivity, improper samples, low viral load, or variable viral shedding (24). When transmitting samples containing viral RNA, it's essential to consider the distance from the sampling site to the testing center since viral RNA is more unstable than deoxyribonucleic acid (DNA). Improper handling and storage of samples during transportation may lead to false-negative results (25-27).

Successful amplification of the first target can be verified by obtaining a positive signal from the secondary target, ensuring that clinical specimens are amplified and detected accurately (28). Normalization is a critical step in quantitative PCR (qPCR) analysis, as it compensates for any changes in sample purity and concentration introduced during the sample preparation phase. These variations can be standardized by normalizing to an internal control (IC) (29-31). To detect SARS-CoV-2, multiplex RT-PCR assays can use any combination of four viral genes (RdRp, E, N, and S genes) according to the World Health Organization's guidelines. Additionally, the Centers for Disease Control and Prevention recommends incorporating primers and probes targeting the human internal/reference RNase P (RP) gene (32).

In the development of commercial diagnostic kits, an approved endogenous IC plays an essential role. The target gene for this type of control is usually a gene whose expression is not affected by the infection in human samples (33). The IC, whether DNA or RNA, is added to the sample during PCR or qPCR setup to ensure that the amplification technique has been performed without any complications. The IC template can also be introduced into the sample before the extraction procedure to further control for extraction. While DNA IC is more stable than RNA IC, using RNA IC to assess the RT-PCR process gives users more confidence in the test procedure because it has the same level of stability as the test case (28).

In this study, we present a performance-based analysis of the commercial Pishtaz Teb kit used in the laboratories of Mashhad University of Medical Sciences and the Geneova kit based on different IC materials. Our study aims to investigate the effect of different ICs on the rate of reporting false negative results in patients with clinical symptoms, a high-resolution computed tomography (HRCT) showing lung involvement, and a negative initial RT-PCR COVID test result.

## Materials and Methods

### Sample Collection

From 2021 to 2022, we conducted a random selection of 162 swab samples from patients exhibiting clinical symptoms of COVID-19 with positive HRCT results. These patients had previously tested negative for SARS-CoV-2 and the swabs were taken from either the nasopharyngeal area or a combination of the nasopharyngeal and oral regions. These samples were sent to the laboratory at Ghaem Hospital in Mashhad, Khorasan Razavi Province, IR Iran, for coronavirus SARS-CoV-2 detection. The results of biochemical and hematology tests of patients were also extracted and analyzed. The protocol of this study has been confirmed by the Ethics Committee of Mashhad University of Medical Sciences. (Code: IR.MUMS.MEDICAL.REC.1400.597)

**Fig. 1.**
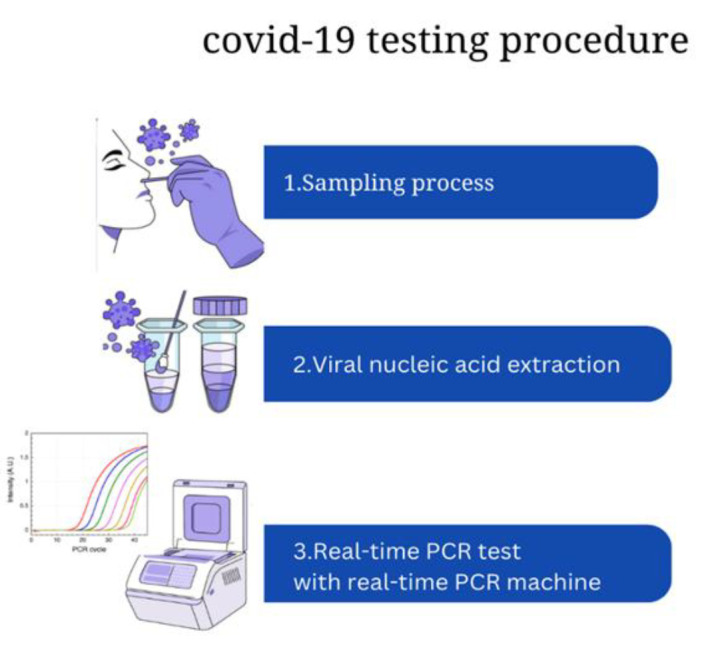
Summary of the clinical and diagnostic method for the diagnosis of COVID-19

### Selection of kits

In this study, two commercially available RT-PCR kits were selected for evaluation: the Pishtaz Teb (COVID-19 One-Step RT-PCR) (Tehran, Iran) and Geneova (GA SARS-CoV-2 One-Step RT-PCR Kit) (Tehran, Iran). The selection was based on their official approval by the Iranian Ministry of Health, high usage in clinical practice, and compatibility with routine RT-PCR workflows. These criteria ensured that the comparison reflected real-world diagnostic conditions and provided insight into the performance of kits commonly used in the national healthcare system.

### Nucleic acid extraction

The whole testing process was shown in Figure 1. The swabs were placed in Virus Liquid Transport Medium (VTM) and transported to the laboratory in a cold box. We maintained the samples for no longer than 8 hours until nucleic acid extraction. Prior to RNA extraction, we vortexed the collection tubes and combined 200 μL of VTM with 10 μL of proteinase K and 200 μL of buffer in 2 mL tubes. We used the Behgene kit (Tehran, Iran) following Iran's guidelines for total nucleic acid extraction.

### Diagnostic Test

RT-PCR test conducted with QIAGEN Rotor-Gene (Germany) implementation . The TaqMan Real-Time PCR method was utilized in both kits. These kits contain different IC materials, for their ability to detect false negative results. RT-PCR analysis was performed on all samples initially tested negative using a commercial kit with human RNaseP target as a DNA IC. The Pishtaz Teb kit was used, which employs a dual-target gene method that targets protected virus genomic sequences of RdRp and N protein nucleocapsid, according to the manufacturer's instructions. Subsequently, the Geneova kit was targeted human RNaseP as an RNA IC to retest the samples. The amplification curves of viral and reference genes were used to evaluate the results, with a positive cut-off value established at a cycle threshold number ≤40, based on a sigmoidal curve. For each detection channel and reaction mixture, negative control for contamination testing and positive control were used. Patients who met the criteria were considered positive. 

### Real-time PCR Analysis

- **Positive and Negative Control Analysis**

RT-PCR was performed using S, ORF-1, RDRP, and N viral targets and human RNaseP as an IC. Positive controls were used to verify the storage method for the reaction mixture and the correct working of the primer and probe. The negative control analysis of COVID-19 PCR was also examined, with all HEX, FAM, TEXAS RED, and CY5 channels assessed.

- **Confirmation Test**

A confirmation test was performed using Geneova GA SARS-CoV-2 kit with four different channels: FAM to detect N region, TEXAS RED to detect S region, CY5 channel to detect the ORF1a region, and HEX to detect RNase P gene as the IC. All reactions were performed in duplicate for four genes. The samples were processed following the manufacturer's instructions, and the results were analyzed using the Rotor-Gene Q software.

### Data Analysis

The compatibility of the Pishtaz and Geneova kits in terms of diagnosing infection will be analyzed using SPSS 18. To compare qualitative variables, the Chi-square test was used, quantitative data was expressed as mean ± standard deviation (SD), and Mann-Whitney was used to compare the means between the groups. P< 0.05 was set as the significant difference.

**Table 1:** Comparison of Two RT-PCR Kits for Detection of SARS-CoV-2

Kit name	Target gene	Internal control	Diagnosis method	Amplification cycle	Volume of RNA	Cut off CT
Pishtaz	RdRp/N protein nucleocapsid	DNA internal control (RNaseP)	TaqMan Real-Time PCR	45	10 μL	≤40
Geneova	N/S/ORF1a	DNA internal control (RNaseP)	TaqMan Real-Time PCR	46	10 μL	≤40

## Results

### Sample Characteristics

**Fig. 2:**
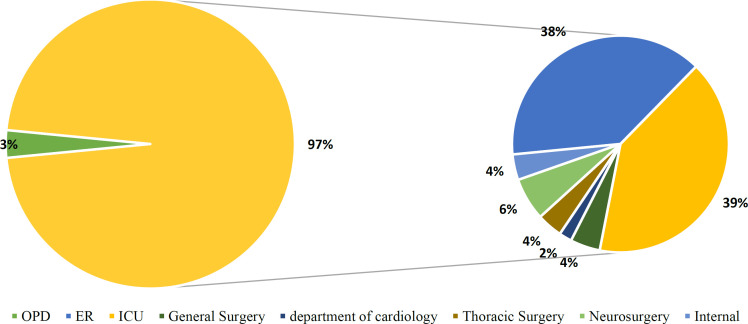
**Distribution of patients based on their department in Mashhad's Ghaem Hospital. **Outpatient Department (OPD) constituted 3% of the total population while hospitalized patients made up 97%. Among the hospitalized patients, Emergency room (ER) accounted for 38%, followed by Intensive care unit (ICU) with 40%, General surgery with 4%, Department of cardiology with 2%, Thoracic surgery with 4%, Neurosurgery with 6%, and Internal disease department with 4%.

A total of 162 SARS-CoV-2 negative samples were collected from patients of different departments of Mashhad's Ghaem Hospital, with an average age of 61.9 years (ranging from 2 to 95 years). Of these, 86 were male and 76 were female (53.1% and 46.9%, respectively), and 5 were outpatients (about 3% of the total population), while 157 (97%) were hospitalized patients (Figure 2).

### Real-time PCR Analysis

Positive control

A sigmoid curve in each channel and a threshold cycle (CT) value less than 35 confirmed that all the reaction components were functioning correctly. Positive controls for this study had sigmoid curves, and the CT was appropriate. Otherwise, the reaction was repeated.

Negative control

All the previously mentioned channels showed a smooth curve, indicating a sigmoid signal curve. A CT value below 40 indicated contamination in the negative control. This study did not reveal any contamination or cross-contamination.

### Internal Control Comparison

**Fig. 3:**
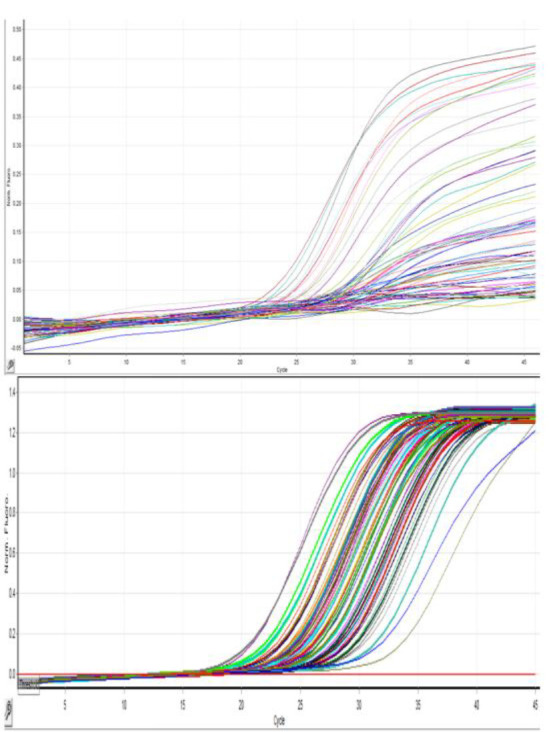
A. Amplification curves of the internal control from the first run of the Geneova kit. B. Amplification curves of the internal control from the first run of the Pishtaz Teb kit.

Initially, the quality of virus extraction was checked using the Pishtaz Teb kit ROX channel. The obtained graphs from the IC were sigmoid. Further evaluation of these samples with the Geneova diagnostic kit showed that only in 44% of cases, the IC of the Pishtaz Teb kit matched with the Geneova diagnostic kit. In the remaining cases, the HEX channel related to the IC of the Geneova kit was negative. Negative cases were reported for re-sampling (Figure 2, 3). Only one patient out of 162 negative samples was positive after re-assay with the Geneova kit. The data did not indicate any relationship between sex and positive IC results (P = 0.6). Men accounted for 51.2% and women accounted for 48.8% of the sigmoid IC curves in the 80 participants. Men accounted for 54.9% and women accounted for 45.1% of the total 82 non-sigmoid curves. In all departments, 49.7% of the 157 hospitalized patients had sigmoid IC compared to 50.3% who did not (P = 0.51 exact Fischer).

CT indicates when a reaction's generated fluorescence rises above the background level and crosses the threshold. The measurable amplicon product of the early exponential phase of the reaction is produced at the threshold cycle. 

### Biochemical and Hematological Test Results

The study included an observation of biochemical and hematological tests requested for 167 patients across different departments. This examination is divided into two groups based on IC graph validation (OK is related to correct representation of IC and Not OK is related to incorrect form of IC). As the results are related to patients with various diseases, some of the minimum and maximum values of the stated parameters may be attributed to other conditions and not necessarily related to COVID-19. However, the examination of average values expressed in Table 1 does not indicate significant changes related to the presence of the coronavirus in the body, which can be explained by the fact that the examined samples are negative for Covid-19.

One notable correlation between the IC and the biochemical and hematological results was found in the Bs test (P ≤ 0.05). It is important to note that clinical symptoms may confirm COVID-19 even when the test is negative. Given that most of the participants in this study had clinical symptoms that suggested COVID-19, this possibility cannot be ruled out (Table 2). 

**Fig. 4:**
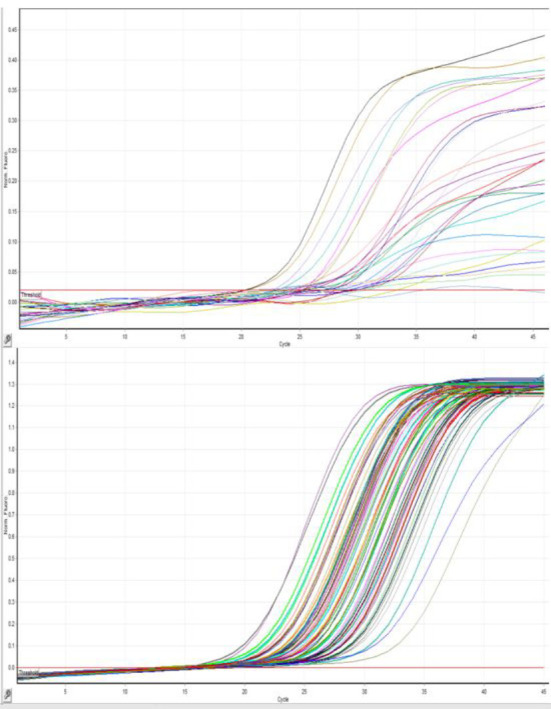
A. Amplification curves of the internal control in the second run using the Geneova kit. B. Amplification curves of the internal control in the second run using the Pishtaz Teb kit.

**Table 2:** Biochemical and hematological tests requested for study participants. (* Mann-Whitney Test)

	IC	N	Median(IQR)*	P-value
BS	Ok	50	124(100-170)	0.02
	Not ok	59		
Urea	Ok	77	49(31-86.7)	0.5
	Not ok	79		
Creatinine	Ok	77	1.1(0.8-1.5)	0.36
	Not ok	79		
ALT	Ok	59	22(14.7-45.2)	0.04
	Not ok	68		
AST	Ok	62	30(20-45.5)	0.73
	Not ok	71		
ALP	Ok	59	325(181.5-360.7)	0.46
	Not ok	71		
Bili t	Ok	54	0.7(0.5-1.4)	0.38
	Not ok	65		
Bili d	Ok	54	0.3(0.2-0.6)	0.21
	Not ok	65		
CRP	Ok	65	77(25-153.7)	0.73
	Not ok	70		
LDH	Ok	34	672(473-1011.2)	0.89
	Not ok	44		
RBC	Ok	73	3.9(3.3-4.5)	0.77
	Not ok	78		
WBC	Ok	74	10.4(7.8-13.5)	0.34
	Not ok	78		
PLT	Ok	74	178(115-252)	0.66
	Not ok	75		
PT	Ok	68	15.5(13.2-18)	0.95
	Not ok	70		
PTT	Ok	67	30(26-35)	0.49
	Not ok	70		
ESR	Ok	61	36(14.2-84.7)	0.05
	Not ok	59		

## Discussion

In this study, the effect of using alternative IC materials on the accuracy of SARS-CoV-2 detection kits was investigated. The study included patients with clinical symptoms and a negative COVID-19 test result, but with lung involvement indicated by a HRCT. The aim was to determine if the use of alternative IC materials could reduce false-negative results. The results showed that only one patient out of 162 negative samples was positive after retesting with the Geneova kit. The positive and negative controls consistently produced the expected results. However, the Geneova IC showed a match with the Pishtaz Teb kit in only 44% of cases, and the Geneova kit had a higher C_T_ value for the IC compared to the Pishtaz Teb kit.

The results of this study are consistent with previous research indicating that proper quality control measures are essential for obtaining accurate results in SARS-CoV-2 RT-PCR testing. Zhang et al. have shown that assessing RNA levels of a housekeeping gene, such as ribonuclease P/MRP component p30, is necessary for reliable results (34). The current study further emphasizes the importance of using appropriate IC materials to ensure accurate diagnoses.

In addition to using appropriate quality control measures, selecting reliable diagnostic kits with high sensitivity and specificity is crucial for reducing false-negative results and accurately identifying COVID-19 patients (35). This decrease in IC can also occur in patient samples, leading to false negative results in cases with a lower viral load or early stages of the disease (28). Possible false negative results may occur as a result of patient recovery from COVID-19, a hospital visit for another reason, or other illnesses that cause the patient to go to the hospital. The Geneova kit in this study showed a lower degree of match with the Pishtaz Teb kit, and had a higher C_T_ value for the IC, which could be due to RNA instability compared to DNA, indicating RNA degradation during the experimental period. This underscores the need for careful selection of ICs to mitigate the risk of false-negative results, particularly in cases with a low viral load or early stages of the disease.

The current study highlights the need for further investigation into the significance of the BS test in relation to the IC, as the increase in BS may be due to COVID-19 disease or other factors. This is consistent with previous research indicating that BS can be a useful indicator of disease severity (36, 37) and even mortality rates (38), among COVID-19 patients. It could be possibly due to the virus's affinity for Angiotensin I-Converting Enzyme type 2 (ACE2) receptors in pancreatic cells that regulate blood sugar (39). Elevated levels of ALT and AST have been reported in COVID-19 patients. These elevations may be due to secondary illnesses including systemic inflammation, hypoxia, or drug-induced liver damage. COVID-19 can induce acute kidney injury, especially in critically ill patients. Of particular note, elevated serum creatinine and BUN are not only indicators of renal impairment but also strong predictors of poor prognosis. One of the hallmark findings in COVID-19 patients is lymphopenia (reduced lymphocyte count), which is observed in most hospitalized cases. Lymphopenia reflects the depletion of immune cells due to viral invasion and immune system exhaustion. Studies show lower lymphocyte counts are associated with severe disease and poor outcomes. CRP is a nonspecific acute-phase reactant that rises in response to inflammation. Elevated CRP levels are a consistent finding in moderate to severe COVID-19 and can be a marker for disease activity and cytokine storm (40).To our knowledge this is the first study to investigate the importance of selecting reliable diagnostic kits with high sensitivity and specificity to reduce false-negative results. These findings provide further evidence that the selection of appropriate ICs is crucial to ensure accurate diagnoses, particularly in cases with low viral load or early stages of the disease. Further research is needed to understand the significance of hematological tests such as ESR in relation to ICs and to continue improving the accuracy of COVID-19 diagnostic tests.

## Conclusion

After repeating the test with a different IC material, only one patient was found to be positive. Although it was expected that testing samples with the same IC material would increase confidence in the extraction process and molecular testing, no significant difference was observed between the two kits in this study, possibly due to the small sample size. However, the results obtained with the Geneova kit demonstrated a higher degree of accuracy in correlation with the symptoms of the patients, which suggests that it may be more reliable than the Pishtaz Teb kit. Therefore, it can be concluded that the choice of a suitable diagnostic kit with high sensitivity and specificity, along with appropriate quality control measures, is crucial for accurate detection of SARS-CoV-2 and effective management of COVID-19 patients.

## Data Availability

The data supporting the results of this study are available upon request from the corresponding author.
